# Evolving care pathways for women with migraine in Italy: results from a national survey

**DOI:** 10.3389/fneur.2026.1755791

**Published:** 2026-03-02

**Authors:** Gianni Allais, Piero Barbanti, Mario Cepparulo, Sabina Cevoli, Cinzia Finocchi, Fabio Frediani, Nicoletta Orthmann, Paola Di Fiore

**Affiliations:** 1 Department of Surgical Sciences, Women's Headache Center, University of Turin, Turin, Italy; 2 Headache and Pain Unit, IRCCS San Raffaele, Rome, Italy; 3 San Raffaele University, Rome, Italy; 4 Teva Italia/SE Hub, Milan, Italy; 5 Programma Cefalee e Algie facciali, IRCCS Istituto delle Scienze Neurologiche di Bologna, Bologna, Italy; 6 Struttura Complessa di Neurologia, Ospedale San Paolo, ASL 2 Savonese, Savona, Italy; 7 Headache Center, Neurology and Stroke Unit, S. Carlo Hospital, ASST Santi Paolo Carlo, Milan, Italy; 8 Fondazione Onda, Osservatorio nazionale sulla salute della donna e di genere ETS, Milan, Italy; 9 ASST Fatebenefratelli Sacco, Milan, Italy

**Keywords:** care pathways, migraine, neurologist, survey, woman

## Abstract

**Background:**

Migraine predominantly affects women with prolonged and more severe episodes that severely compromise their quality of life. Despite its burden, diagnostic-therapeutic pathways specifically designed for women are currently lacking, and care for women with migraine remains fragmented. By expanding the results obtained from a recent Delphi consensus study, we aim to identify key unmet needs and propose expert-driven recommendations to address female-specific migraine care in Italy.

**Methods:**

We conducted a national survey in which the items used in the previous Delphi study were administered to a broader cohort of Italian healthcare professionals (HCPs). The survey was administered to 125 HCPs; of those, 92% were neurologists.

**Results:**

Diagnostic delays, insufficient multidisciplinary collaboration, and heterogeneous care emerged for women with migraine across Italy. Respondents endorsed parental awareness and socio-behavioral approaches for pediatric patients, careful evaluation of thrombotic risk for contraceptive choice, systematic assessment of migraine history and new-onset migraine during pregnancy and breastfeeding, and appropriate selection of hormone replacement therapy during menopause. Close monitoring of migraine symptoms is encouraged in oncological patients and women undergoing assisted reproduction, although evidence and guidelines for these patient subsets are currently lacking. Across all settings, the neurologist emerged as the central medical reference figure from adolescence onward, working in close collaboration with gynecologists, pediatricians, and oncologists as appropriate. Strengthening continuity of care, enhancing multidisciplinary collaboration, expanding professional training, and promoting awareness initiatives are considered the key strategies to optimize migraine care in women.

**Conclusions:**

Based on the respondents' answers, we propose practical frameworks that outline migraine care pathways tailored to specific female life stages and health conditions. Efforts should prioritize the design of targeted studies to overcome the identified evidence gaps and pave the way for more structured models.

## 1 Introduction

Migraine is a highly underestimated and burdensome neurological disease. Women are three times more affected than men and face more severe attacks, with negative implications on everyday activities (1–8). Over the past three decades, migraine prevalence, incidence, and disability have risen markedly among women of reproductive age (9). Fluctuations in estrogen levels are recognized as key contributors to female migraine pathophysiology, further complicating diagnosis and management of female patients in different life phases, such as menarche, pregnancy, and menopause (1, 2).

In Italy, individuals with migraine exhibit low consultation rates with both general practitioners (GPs) and specialists, regardless of gender; a considerable underuse of effective pharmacological options has also been observed (10–15). Nonetheless, women consistently present with a more severe clinical profile compared to men, with longer attacks and higher frequency of moderate-to-severe pain. They report a higher number of missed workdays and lower participation in social activities, yet they are more likely to attend work despite active migraine symptoms. Consequently, women with migraine exhibit a lower quality of life than men, largely due to the disruption of daily functioning (16, 17). Recent findings from the Italian National Migraine Registry (I-GRAINE) offered additional real-world evidence, identifying the typical Italian patient with migraine as an employed woman in her mid-forties, often physically inactive and affected by sleep disturbances (18). Although women bear a great migraine burden, they often fail to receive appropriate medical care. The perception of migraine as a predominantly female disorder, frequently accompanied by social stigma, contributes to its underrecognition and undertreatment (9, 10, 16). Given the specific and evolving needs of female patients, particularly during hormonally sensitive life stages or in the presence of comorbidities, *ad hoc* approaches are pivotal to the effective management of migraine in women.

A recent Delphi consensus by Cevoli et al. (19) reviewed current practices in managing female migraine within the Italian healthcare system, identifying key challenges and unmet needs. The study provides expert-driven recommendations to guide the development of standardized, gender-sensitive care models, with strategies tailored to female-specific needs across life stages and comorbid conditions. The panel emphasized the importance of raising awareness of migraine and its impact; comprehensive education for healthcare professionals (HCPs) and multidisciplinary collaboration were recommended to improve recognition, diagnosis, and treatment of this insidious disease (19).

Building upon these previous findings, the present national survey was conducted to expand the perspective beyond that of a selected expert panel by capturing the views of a wider range of specialists involved in migraine care. This approach enables a more accurate evaluation of the current management of women with migraine within the Italian healthcare system, identifying unmet needs and generating further insights for the implementation of optimized diagnostic and therapeutic pathways. The outcomes of this survey may also serve as a valuable reference for similar investigations in other international contexts.

## 2 Materials and Methods

### 2.1 Survey promotion and administration

This survey was promoted by a panel of six headache specialists, members of the Italian Neurological Association for Headache Research (Associazione Neurologica Italiana per la Ricerca sulle Cefalee, ANIRCEF). The survey was administered in July 2024; it was distributed via email, accompanied by a cover letter, to 200 HCPs with certified expertise in headache across the Italian national territory. Results were collected and data were analyzed in September 2024.

Up to 125 participants answered the survey (response rate: 63%). Of these, 85 respondents provided complete information on specialty and geographic distribution.

### 2.2 Survey structure

The survey comprised 65 items focusing on disease perception, diagnosis and management of women with migraine, as well as strategies to improve the current care while considering the varying needs across different life stages and health conditions. The questionnaire was derived from a previously validated Delphi study (19). The characteristics of panelists involved in the original Delphi study were comparable to those of the current survey respondents in terms of migraine expertise and specialty distribution, and no further validation was deemed necessary. As in the original Delphi study, most of the items (n=57 statements) were assessed using a 4-point Likert scale to evaluate the level of agreement, with response options ranging from 1 (“Fully Disagree”) to 4 (“Fully Agree”). *A priori* consensus criteria were applied, with agreement defined as ≥67% of respondents selecting either “Partially Agree” or “Fully Agree”. In addition, the survey also included questions that required respondents either to select from multiple options or to rank available choices. (19).

## 3 Results

### 3.1 Participants

Among the 85 HCPs who reported their specialty and geographic distribution, respondents were located across 13 Italian regions (68.2% from Northern Italy, 12.9% from Central Italy, and 18.8% from Southern Italy). The majority were neurologists (92%), with smaller proportions of other specialists, including clinical pharmacologists and internists (7%), who are involved in migraine care within specialized centers. Within the neurologist subgroup, 81% reported more than 10 years of experience in migraine management and 67% were based in hospitals or in migraine-specialized centers.

### 3.2 Survey results

The complete results of the survey are presented in Supplementary Table S1 in Supplementary Materials. The following sections summarize the main findings.

### 3.3 Women's perception of migraine

Respondents believe that most women consider migraine either as a symptom of other disorders (59/125; 47.2%) or as a pathology (50/125; 40.0%), with a smaller proportion regarding it as a normal recurring condition (16/125; 12.8%; Q1). Most respondents (91/122; 74.6%) agreed that women with migraine are uncertain about where to seek appropriate care (Q2). The survey results confirm the substantial burden of migraine in women (Q3), with 93.3% (111/119) of respondents acknowledging its profound social impact, including reduced quality of life, impaired relationships, and the need for support, and 87.2% (102/117) recognizing significant work-related consequences (presenteeism, absenteeism and limited professional opportunities; Q4). Stigma also emerged as a critical concern (94/119; 81.0%, Q5).

### 3.4 Diagnosis and management of migraine

According to 42.1% of respondents (48/114), women typically wait more than 5 years after symptom onset before undergoing their first medical assessment (Q6). Of even greater concern, 37.7% (43/114) reported that a diagnosis is made only after at least 1 year following the initial medical evaluation, and 14.0% (16/114) indicated diagnostic delays extending beyond 5 years (Q7). Access to headache specialists is also delayed: only 3.5% of respondents (4/114) reported that patients are seen within 1 month, whereas the majority indicated longer waiting times (6–12 months: 43/114; 37.7%; over 1 year: 21/114; 18.4%; Q8). Overall, the current care is widely judged as insufficient (86/114, 75.4%; Q9), characterized by lack of multidisciplinarity (97/114; 85.1%; Q10), lack of continuity of care (86/113; 76.1%; Q11), insufficient territorial medical references (84/113; 74.3%; Q12). The survey responses clearly reveal the heterogeneous and non-standardized nature of migraine care across Italy (103/113; 91.2%; Q13).

In most cases, respondents believe that the initial point of contact is a HCP other than a neurologist, most commonly the pediatrician for children (94/110; 85.5%), the GP for adolescents (80/110; 72.7%) and adults with menstrual or menstrual-related migraine (66/110; 60.0%), the gynecologist for adult women with migraine who are pregnant/breastfeeding (91/110; 82.7%) or are undergoing contraceptive therapy (85/110, 77.3%), or oncologist for women with migraine undergoing oncological treatment (99/110; 90.0%; Q14). Regardless of the current pattern of care, most respondents agreed that pediatric patients with migraine should ideally be managed by their pediatrician (71/109; 65.1%), while 53.2% (58/109) also identify the child neuropsychiatrist as a key reference figure for this age. From adolescence onward, respondents regard the neurologist as the principal point of reference for all female patients with migraine, with agreement across age life phases and health conditions ranging between 68.8% (75/109) and 88.1% (96/109). In patients with comorbidities or requiring specialized care, additional specialists, such as gynecologists for pregnant or breastfeeding women (84/109; 77.1%) and oncologists for women undergoing oncological treatments (82/109; 75.2%), should complement the role of neurologists. By contrast, non-clinical HCPs (e.g., pharmacists, psychologists) are generally not regarded as primary points of reference in migraine management (Q15).

### 3.5 Management of pediatric and adolescent patients with migraine

The survey highlighted the importance of psychosocial, behavioral, and educational aspects for the optimal management of migraine in pediatric and adolescent female patients with migraine. Most respondents (104/109; 95.4%) agreed that social factors potentially triggering migraine attacks should be thoroughly investigated (Q16), and 92.5% (99/107) endorsed socio-behavioral interventions as the preferred first-line approach to prevent or reduce episodes (Q17). A combined socio-behavioral and pharmacological strategy was supported by 93.5% (100/107) of respondents when socio-behavioral measures alone are insufficient (Q18). According to 75.5% (80/106) of respondents, the psychologist should be consulted only in cases of suspected stress or anxiety (Q19), whereas 90.5% (95/105) of respondents endorsed psychiatrist involvement when a psychopathological disorder is suspected (Q20). The need to increase parental awareness of migraine and its symptoms strongly emerged as a unanimous priority (105/105, 100%; Q21).

**Figure 1:**
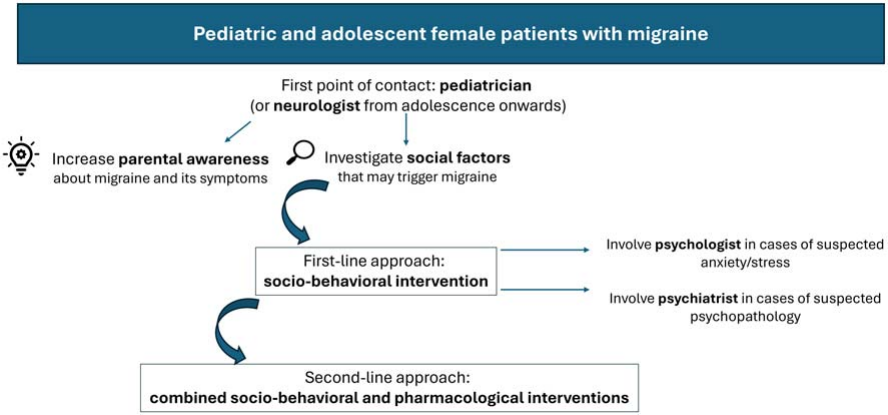
Framework for improved diagnostic-therapeutic care pathways in pediatric and adolescent female patients with migraine, as emerged from the survey responses.

Figure 1 presents a flowchart outlining the core components of an optimized diagnostic–therapeutic care pathway for pediatric and adolescent female patients with migraine, developed on the basis of the survey responses.

### 3.6 Management of women with migraine during adulthood

The management of migraine in adult women has been analyzed in relation to medical needs that may arise during adulthood (i.e., menstrual migraine, contraceptive use, fertility or oncological treatments) as well as physiological transitions, including menopause.

#### 3.6.1 Women with menstrual migraine or menstrual-related migraine

Fewer than half of respondents (45/102; 44.2%) agreed that specialist consultation should be limited to cases of prolonged or severe symptoms, indicating the need for earlier involvement (Q22). The majority (78/102; 76.5%) indicated that neurologists/headache specialists should be primarily involved in migraine care in these patients (Q23).

#### 3.6.2 Women requiring or undergoing contraceptive therapy

Nearly all respondents agreed that migraine and the presence of aura must be carefully considered when prescribing contraception (100/101; 99.0%; Q24), with the choice of contraceptive therapy being made according to ischemic risk factors (95/99; 96.0%; Q25). Neurologist/headache specialist involvement is essential for diagnosing migraine with aura (74/97; 76.3%; Q26). Most respondents (86/96; 89.6%) supported routine thrombotic risk assessment before contraceptive choice (Q27).

#### 3.6.3 Pregnant or breastfeeding women

Respondents unanimously agreed that migraine presence should be assessed at the first gynecological visit (96/96; 100%; Q28); in addition, the gynecologist should pay attention to any new-onset migraine during pregnancy (96/96; 100%; Q29). Overall, respondents supported the use of anti-migraine medications, with 71.6% (68/95) endorsing the administration of safe therapies during pregnancy or breastfeeding (Q30). To ensure optimal care, most respondents considered the involvement of the neurologist/headache specialist as necessary (91/94; 96.8%; Q31). Similarly, respondents also recognized the use of acupuncture as complementary migraine treatment (87/94; 92.6%; Q32).

#### 3.6.4 Women undergoing assisted reproduction

According to 97.8% (92/94) of respondents, the presence of migraine needs to be assessed prior to initiating assisted reproduction (Q33), with 86.2% (81/94) endorsing the potential use of alternative, lighter hormonal stimulation protocols to reduce migraine symptoms (Q34). Consultation with the neurologist/headache specialist was strongly emphasized (92/94; 97.9%; Q35); psychological support was also advocated by 87.2% (82/94) of respondents (Q36).

#### 3.6.5 Women in menopause

All respondents (94/94; 100%) agreed that hormone replacement therapy (HRT) should be chosen after careful clinical evaluation (Q37), and that the peri-menopausal phase warrants particular attention, as hormonal fluctuations during this period are associated with an increased risk of migraine attacks (Q38). Accordingly, new-onset migraine during menopause or HRT should always be investigated (94/94; 100%; Q39) and the neurologist/headache specialist should be consulted for optimal treatment of this patient subset (91/94; 98.9%) (Q40).

#### 3.6.6 Women with cancer undergoing oncologic treatment

All respondents (93/93, 100%) agreed that attention should be paid to any worsening of migraine symptoms occurring during anti-cancer treatments (Q41). As with other adult female subgroups, the neurologist or headache specialist was identified as the central figure in migraine management, working in close collaboration with the oncologist (92/93; 98.9%; Q42).

**Figure 2:**
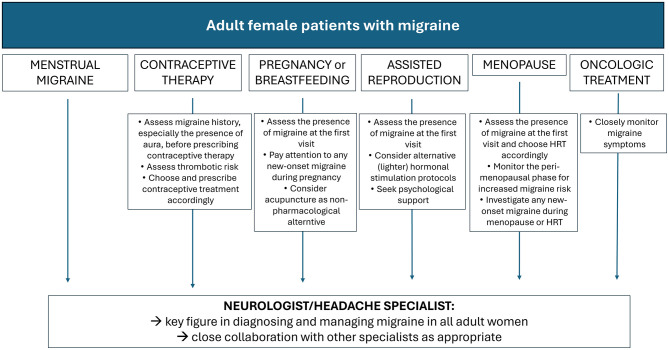
Frameworks for improved diagnostic-therapeutic care pathways in adult women, as emerged from the survey responses.

Figure 2 presents a flowchart outlining the core elements toward optimized diagnostic-therapeutic care pathways for adult women with migraine, considering their specific needs as emerged by the survey responses.

### 3.7 Strategies to improve migraine management in women

The need to guarantee continuity of care for women with migraine across all life stages strongly emerged from the survey's responses (92/93; 98.9%; Q43); management of patients should take place either in headache centers/specialized clinics (85/93; 91.4%; Q44) or through community-based care (85/93; 91.4%; Q45), while 97.9% (91/93) of respondents advocated for a shared-care model between headache centers and territorial facilities (Q46). The caregiver was also recognized as playing a key role in migraine management, as reported by 80.7% of respondents (75/93; Q47).

Digital health solutions were also considered relevant for strengthening collaboration among centers, including the use of a dedicated digital platform to facilitate communication and information sharing (80/93; 86.0%; Q48), and telemedicine (79/93; 84.9%; Q49).

Continuous education on migraine is widely regarded as essential across all healthcare levels, with strong support for mandatory training for pharmacists (79/93; 84.9%; Q51), GPs and pediatricians (89/91; 96.7%; Q54), and specialists (83/91; 91.2%; Q57). Nearly all respondents (89/91; 97.8%) highlighted the need for more evidence to guide migraine care in specific settings (i.e., women undergoing assisted reproduction or oncological treatments; Q59).

Raising awareness of migraine should also be prioritized, with broad support for the implementation of diverse initiatives (e.g., the use of social media platforms, the number of “headache awareness days”, the availability of questionnaires in pharmacies, agreement >85%; Q60–Q63). Nearly all respondents (93.4%; 91/91) agreed on the usefulness of providing women with a pamphlet outlining the significance of symptoms and the different phases of the patient journey (Q64). Respondents also identified other relevant themes, such as medical reference figures for migraine care (76/91; 83.5%), symptom neglect (61/91; 67.0%), socio-economic disease burden (51/91; 56.0%; Q65).

**Figure 3:**
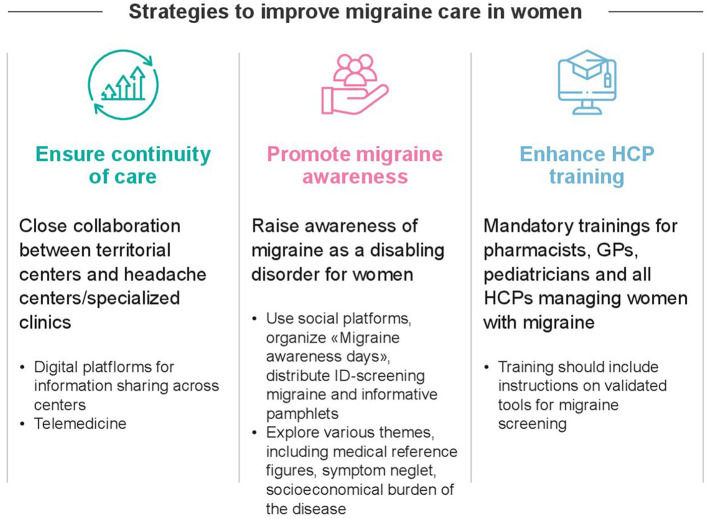
Proposed strategies to improve current management of women with migraine, as emerged from the survey responses.

Figure 3 illustrates the fundamental pillars and correspondent practical strategies to improve the current management of migraine in women, based on the respondents' opinions.

## 4 Discussion

Building on the results from the earlier Delphi study (19), this national survey offers a broader perspective on the current management of migraine in women across Italy. Based on the responses, practical frameworks have been proposed for improved diagnostic-therapeutic care pathways tailored to female-specific needs.

### 4.1 Perception of women's migraine burden and current care

The high burden of migraine among women in Italy has been consistently observed in both national and international studies (4–8, 17). A recent global analysis revealed that Italy was among the countries where the migraine burden remains higher than expected given available resources (20). This pattern has been largely confirmed by the surveyed HCPs, who also acknowledged the stigma surrounding migraine in women. Respondents also noted that many women misinterpret migraine symptoms and fail to recognize them as a genuine disease, contributing to delays in seeking professional care.

Despite the detrimental impact of migraine on the Italian female population, current care is far from being optimal. Respondents highlighted the lack of multidisciplinary approaches, poor continuity of care, and the absence of clear medical references.

Notably, the results suggest that most women initially consult a range of HCPs other than neurologists, reflecting their uncertainty in navigating the healthcare system. While GPs should serve as the primary point of contact for early recognition and preliminary management of migraine (21, 22), respondents consistently considered neurologists to be the ideal central medical reference for all women from adolescence onward, irrespective of age, comorbidities, or concomitant treatments.

### 4.2 Migraine care in pediatric female patients

Although less common than in adults, migraine still affects a substantial proportion of children and adolescents, with increasing prevalence from childhood to adolescence, particularly among females (23, 24). In pediatric patients, management strategies are generally similar between sexes; however, from adolescence onward, sex-related differences emerge, necessitating preferential involvement of the neurologist. In line with guidelines recommendations (25, 26), respondents have reiterated the importance of lifestyle and behavioral interventions, as well as enhanced education to avoid triggers and reduce headache frequency. Psychological support may be warranted in cases of anxiety or stress; indeed, anxiety and depression may coexist with migraine in children and adolescents (27), potentially contributing to headache persistence (28). Psychiatrists may also play a role in managing pediatric patients with psychiatric comorbidities (29, 30). Pharmacological therapy may also be considered (25, 26) although clinical trials have demonstrated a high placebo response in children and adolescents, leading to a lower overall therapeutic gain from medications (31). The key components of the ideal diagnostic–therapeutic care pathway for female pediatric patients as derived from the survey are summarized in Figure 1.

### 4.3 Migraine care in adult women

During adulthood, women's physiological changes and sex-specific medical conditions influence migraine patterns, thereby necessitating tailored care. Although management strategies are relatively well established for menstrual migraine, menopausal migraine, and migraine in women using contraceptives, they remain largely undefined in contexts such as pregnancy, breastfeeding, cancer therapy, and assisted reproduction. The framework presented in Figure 2 outlines the diagnostic-therapeutic priorities according to female life stages and clinical circumstances, as emerged from the survey responses and discussed below.

#### 4.3.1 Menstrual migraine

Many women with migraine report that their attacks are temporally linked to menstruation (3). In menstrual migraine, the rapid decline in estrogen levels and the rise in prostaglandin levels that precede the menstrual cycle are the key pathophysiological mechanisms triggering the disease (32). Although menstrual migraine is generally treated with the same therapeutic strategies as other migraine types (33, 34), it tends to be more severe and less responsive to standard medications, highlighting the need for novel and more effective therapeutic strategies (32, 35). Unlike the original Delphi study, the results of the present survey suggest that patients with menstrual migraine may be referred to a specialist regardless of symptom duration or severity.

#### 4.3.2 Contraceptive therapy

Contraceptive therapy is widely used among women of reproductive age. However, estrogen-containing formulations should be strictly avoided in women with migraine with aura (36), due to their elevated risk of cerebrovascular and cardiovascular events (37–41); progestin-only contraceptives are therefore preferred (42–44). As such, physicians should assess migraine history, especially the presence of aura, before prescribing any contraceptive method. Although not currently recommended by guidelines, respondents agreed that thrombotic risk should be evaluated. Clinicians who routinely manage patients with migraine may be more aware of this risk. In contrast, guidelines often adopt a broader perspective that may overlook gender-specific considerations. A thorough investigation of the actual thrombosis risk may assist gynecologists in selecting the most suitable contraceptive option according to the individual patient's risk profile, thereby maximizing safety.

#### 4.3.3 Pregnancy and breastfeeding

Migraine should receive special medical attention also in pregnant or breastfeeding women. During pregnancy, migraine is associated with an increased risk of gestational complications, including hypertension, preeclampsia, and vascular events (37). Therefore, gynecologists should systematically assess any history of migraine and evaluate new-onset migraine at the first consultation. In women with established migraine, selected therapies may be used (45); however, potential fetal adverse effects from standard migraine therapies have also been documented (46, 47). In light of these safety concerns, respondents agreed that non-pharmacological approaches, like acupuncture, should be prioritized in pregnant and breastfeeding women (48, 49).

#### 4.3.4 Assisted reproduction

*In vitro* fertilization and embryo transfer are increasingly used in clinical practice for infertility management. These assisted reproductive techniques rely on treatment protocols that induce marked hormonal fluctuations. Given the close relationship between the variation in estrogen levels and migraine, severe attacks may occur during hormonal therapy (50, 51). Indeed, a study found that 28.6% of women undergoing such treatments experienced headaches, which were significantly more frequent among those with migraine, with 82% describing them as severe (52). For this reason, migraine history should be carefully assessed during the initial consultation and before starting treatment. Survey respondents encouraged prioritizing alternative protocols with lighter hormonal stimulation and integrating psychological support into care. However, evidence on the most appropriate strategies to manage migraine in women undergoing assisted reproduction is currently lacking and no specific protocols or interventions are recommended in existing guidelines. Therefore, dedicated research is urgently needed to optimize care in this increasingly common subset of female patients.

#### 4.3.5 Menopause

Although migraine declines with age in most individuals, a rise in prevalence has been documented in women during perimenopause (24, 53–55). HRT, widely used to manage vasomotor symptoms in menopause, may provide beneficial effects for migraine management, although evidence is contrasting (55–57). However, the use of estrogen-based HRT requires caution due to a potentially increased risk of ischemic stroke (58). Therefore, a comprehensive evaluation of migraine history is essential before initiating HRT, which should be chosen in an individualized manner. While the lowest effective dose of estrogen should be prescribed (33), alternative options such as phytoestrogens and continuous progestogen regimens should also be considered (55–57, 59).

#### 4.3.6 Oncologic treatment

There is no literature that informs on how migraine should be managed in women undergoing oncological treatment. This is surprising, as women with breast cancer have higher migraine prevalence compared to the female general population (60). While anti-cancer treatments may worsen migraine pain, to date the only breast cancer therapy proven to affect headache burden is locoregional radiotherapy (60). Notably, there is a complete lack of data regarding the impact of selective estrogen receptor modulators (SERMs) on migraine course. Given the well-established effects of estrogen fluctuations on migraine, it would be clinically valuable to investigate how SERMs influence the frequency and intensity of migraine attacks in oncological patients. Considering such a lack of literature and guidelines, the survey's responses recommended enhanced monitoring of migraine symptoms in patients undergoing oncological treatment. *Ad hoc* research in this field is strongly warranted to guide clinicians in defining best practices.

**Figure 4:**
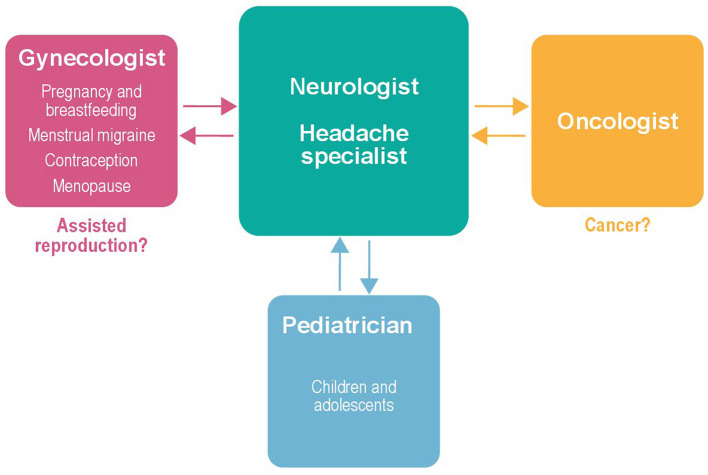
The neurologist as the central medical reference for women with migraine: the need for a multidisciplinary approach and bidirectional collaborations to expand knowledge in special contexts.

Despite the distinct frameworks proposed, a common feature across all patient settings is the central role of the neurologist/headache specialist, who is consistently considered the main reference figure regardless of women's age or comorbidities, to ensure accurate diagnosis and appropriate intervention. In turn, the neurologist/headache specialist should work in a multidisciplinary fashion with other specialists, such as gynecologists, pediatricians, and oncologists, as appropriate. Even if not formally trained in headache medicine, these specialists should be aware of the disabling nature of migraine in women in terms of frequency, clinical burden, and impact on quality of life, to ensure timely recognition and appropriate referral. As the available evidence remains insufficient to define clear standards of care, especially for complex scenarios such as assisted reproduction or comorbid oncological conditions, the neurologist/headache specialist should establish a bidirectional collaboration with the other specialists to share clinical expertise and conduct further research. Figure 4 presents a descriptive model illustrating the central role of the neurologist/headache specialist in female migraine care while establishing multidisciplinary interactions with other relevant specialists, based on patients' needs.

### 4.4 Proposed strategies to improve migraine management in women

To improve migraine care in women, strategies should address three key priorities: (1) ensuring continuity of care, (2) strengthening healthcare professional training, and (3) promoting migraine awareness. Figure 3 summarizes the main strategies emerged from the survey's responses to address these priorities. Continuity of care between community services and specialized headache centers should be reinforced, with digital platforms and telemedicine supporting information exchange and shared management. Regular training is required to enhance knowledge of migraine, its diagnosis, and available treatment options among all HCPs involved in women's care. Involvement of key stakeholders such as healthcare systems and scientific societies is crucial to increase the number of training programs and educational initiatives to enhance HCPs' understanding of migraine in women. Practical steps may include offering national/local workshops led by headache specialists and developing practical tools to support clinical decision-making and encourage adherence to guidelines. Launching multidisciplinary educational campaigns in collaboration with relevant professional societies (e.g., neurology, primary care, gynecology) may also foster inter-specialty collaboration and expand the network of local HCPs equipped to manage migraine effectively. Equally important is the promotion of awareness among patients and the general public, both to strengthen recognition of migraine as a disabling neurological disorder requiring medical attention and to reduce the stigma that continues to surround women with migraine. Patient associations can collaborate with HCPs and policymakers to disseminate accurate, accessible information. Several strategies were perceived as valuable for increasing public awareness, including the use of social media platforms, a greater number of dedicated “headache awareness days”, and the availability of screening tools such as ID-Migraine questionnaires in pharmacies. An informative pamphlet designed for women with migraine could help accelerate diagnosis and promote optimal management by sensitizing patients to migraine symptoms and advising on appropriate HCP consultation. By simultaneously tackling these three key areas, meaningful progress can be made in improving care for women with migraine.

### 4.5 Strengths and limitations

The key strengths of this study are the relatively large and diverse sample of Italian HCPs that responded to the survey. Although the majority of respondents were neurologists, the participation of non-neurologist professionals with proven experience in migraine care provided valuable multidisciplinary input. Future research should aim to involve a broader range of specialties to capture additional perspectives and further strengthen the multidisciplinary approach to migraine care.

In addition to the predominance of neurologists, most respondents were based in Northern Italy, within specialized centers. Consequently, subgroup analyses by specialty, region, practice setting, or years of experience were not feasible, as many subgroups included too few participants to allow for meaningful comparisons.

Additional limitations that may have influenced the findings and their interpretation include the potential for recall bias and the reliance on self-reported perceptions, which are inherently subject to personal interpretation and reporting inaccuracies. Moreover, the survey reflects the perspectives of expert HCPs only and does not incorporate patient views. Future studies would benefit from integrating patient-reported outcomes and preferences to provide a more comprehensive understanding of female migraine care needs.

## 5 Conclusion

At present, shared models of care for women with migraine have yet to be established. Building on the findings of the previous Delphi study, this survey corroborates the urgent need for sex- and age-sensitive strategies to improve diagnosis, treatment, and long-term management of women with migraine, further underscoring the clinical heterogeneity associated with female physiological transitions and the diverse medical needs that may arise across lifespan. While based on Italian HCPs' perspective, the findings may be generalized to other healthcare systems, particularly within Europe. Indeed, the barriers to effective migraine management are not unique to Italy and are widely encountered across European healthcare systems (61–64). Thus, the priorities and proposed care frameworks for women presented in this study may serve as a valuable reference for informing similar initiatives beyond Italy.

The survey responses have been instrumental in drafting *ad hoc* frameworks for tailored migraine management in women. Importantly, all the proposed frameworks are conceptual and derived from expert opinions. Accordingly, they should be interpreted as exploratory models to support discussion and inform future research and care planning. While additional research is required to establish optimal strategies, efforts should now focus on refining and implementing these approaches, with the ultimate goal of reducing disease burden and enhancing the well-being of women affected by this burdensome disorder.

## Data Availability

The original contributions presented in the study are included in the article/Supplementary material, further inquiries can be directed to the corresponding authors.
