# Supplementary material for: Evolving care pathways for women with migraine in Italy: results from a national survey
Source: Front Neurol. 2026 Mar 2;17:1755791. doi: 10.3389/fneur.2026.1755791 (PMC12989344; doi:10.3389/fneur.2026.1755791)
Supplement: Supplementary file 1 [file Table_1.docx]

Supplementary Material

Table S1. Survey results. Responses are presented as % (n) of respondents who expressed partial or full agreement with each statement.

| **Awareness of the condition for patients**  **Q1. In your experience, how does a woman suffering from migraine perceive migraine? (n=125)** | |  |
| --- | --- | --- |
|  | Most frequent answer, % (n) | |
| A symptom of another cause (e.g., hormonal fluctuations, cervical issues, etc.) | 47.2% (59) | |
| A pathology | 40.0% (50) | |
| A recurring normal and non-pathological condition | 12.8% (16) | |

| **General framework, perception of the illness: indicate the degree of agreement to the following statement** | | | | | | | | | |
| --- | --- | --- | --- | --- | --- | --- | --- | --- | --- |
|  | |  | | | Agreement, % (n) | | | | |
| In your experience, the woman with migraine | | Q2. Is aware of having a pathology but does not know whom to turn to (n=122) | | | 74.6% (91) | | | | |
|  |  | Q3. Is subjected to a high burden of the disease in social terms (low quality of life, missed participation in social activities, negative impact on relationships, need for support) (n=119) | | | 93.3% (111) | | | | |
|  |  | Q4. Is subjected to a high burden of the disease in terms of work/economics (high percentage of absenteeism, negative impact on career prospects) (n=117) | | | 87.2% (102) | | | | |
|  |  | Q5. Perceives her symptoms as a stigma (n=114) | | | 81.0% (94) | | | | |
| **Diagnosis of the condition: please answer to the following questions** | | | | |  | | | | |
|  | |  | | | < 1 year, % (n) | | Between 1 and  3 years, % (n) | Between 3 and 5 years, % (n) | >5 years, % (n) |
| In your experience | | Q6. On average, how long between the onset of symptoms and the first medical assessment? (n=114) | | | 8.8% (10) | | 29.8% (34) | 19.3% (22) | 42.1% (48) |
|  |  | Q7. On average, how long between the first medical assessment and the diagnosis? (n=114) | | | 31.6% (36) | | 40.4% (46) | 14.0% (16) | 14.0% (16) |
|  |  |  | | | < 1 month, % (n) | | Between 1 and 6 months, % (n) | Between 6 months and one year, % (n) | Over one year, % (n) |
|  |  | Q8. What is the average waiting time for the first specialized neurological visit at a headache center or with an expert neurologist? (n=114) | | | 3.5% (4) | | 40.4% (46) | 37.7% (43) | 18.4% (21) |
| **General features of current management: Indicate the level of agreement to the following statement** | | | |  | | |  |  |  |
|  | |  | | | Agreement, % (n) | | | | |
| In your experience, the current management of patients with migraine | | Q9. It is insufficient (n=114) | | | 75.4% (86) | | | | |
|  |  | Q10. It is characterized by the lack of multidisciplinary approach (n=114) | | | 85.1% (97) | | | | |
|  |  | Q11. It is characterized by the lack of continuity of care (n=113) | | | 76.1% (86) | | | | |
|  |  | Q12. It is characterized by the lack of medical references at the territorial level (n=113) | | | 74.3% (84) | | | | |
|  |  | Q13. It is heterogeneous and non-standardized in Italy (n=113) | | | 91.2% (103) | | | | |
| **General features of current management: Indicate, for each stage of life, the responses you consider most appropriate** | | | | | | | | | |
| Q14. In your experience, the primary point of reference for a woman experiencing migraine symptoms in various stages of life and specific health conditions is represented by (possible more than one answer) (n=110): | | | | | | | | | |
|  | Pediatric age, % (n) | | Adolescence, % (n) | Adulthood with menstrual or menstruation-related migraine, % (n) | Adulthood while undergoing contraceptive therapy, % (n) | Adulthood, pregnancy and breastfeeding, % (n) | | Menopause, % (n) | Adulthood during oncological treatment, % (n) |
| a. The pharmacist | | 8.2% (9) | 11.8% (13) | 26.4% (29) | 13.6% (15) | 5.5% (6) | | 11.8% (13) | 0% (0) |
| b. The psychologist | | 1.8% (2) | 2.7% (3) | 0.0% (0) | 0.9% (1) | 0.0% (0) | | 0.0% (0) | 2.7% (3) |
| c. The pediatrician | | 85.5% (94) | 22.7% (25) | 0.91% (1) | 0.0% (0) | 0.9% (1) | | 0.0% (0) | 0.0% (0) |
| d. The general practitioner | | 19.0% (21) | 72.7% (80) | 60.0% (66) | 43.6% (48) | 24.6% (27) | | 60.9% (67) | 22.7% (25) |
| e. The child neuropsychiatrist | | 18.2% (20) | 10.0% (11) | 0.9% (1) | 0.9% (1) | 0.0% (0) | | 0.0% (0) | 0.0% (0) |
| f. The neurologist and/or headache specialist | | 9.1% (10) | 24.6% (27) | 47.3% (52) | 46.4% (51) | 49.1% (54) | | 63.6% (70) | 44.6% (49) |
| g. The gynecologist | | 2.7% (3) | 5.5% (6) | 53.6% (59) | 77.3% (85) | 82.7% (91) | | 35.5% (39) | 2.7% (3) |
| h. The oncologist | | 0.0% (0) | 0.0% (0) | 0.0% (0) | 0.0% (0) | 0.0% (0) | | 0.0% (0) | 90.0% (99) |
| i. Other | | 3.6% (4) | 4.6% (5) | 2.7% (3) | 0.9% (1) | 0.9% (1) | | 1.8% (2) | 0.9% (1) |
| **Q15. Desirable management: Indicate, for each stage of life, the responses you consider most appropriate** | | | | | | | | | |
| In your experience, the primary point of reference for a woman experiencing migraine symptoms in various stages of life and specific health conditions should be represented (possible more than one answer) (n=109): | | | | | | | | | |
|  | | Pediatric age, % (n) | Adolescence, % (n) | Adulthood with menstrual or menstruation-related migraine, % (n) | Adulthood while undergoing contraceptive therapy, % (n) | Adulthood, pregnancy and breastfeeding, % (n) | | Menopause, % (n) | Adulthood during oncological treatment, % (n) |
| a. The pharmacist | | 0.9% (1) | 1.8% (2) | 1.8% (2) | 1.8% (2) | 0.9% (1) | | 0.9% (1) | 0.9% (1) |
| b. The psychologist | | 7.3% (8) | 14.7% (16) | 3.7% (4) | 2.8% (3) | 2.8% (3) | | 4.6% (5) | 6.4% (7) |
| c. The pediatrician | | 65.1% (71) | 28.4% (31) | 0.9% (1) | 0.0% (0) | 0.9% (1) | | 0.0% (0) | 0.0% (0) |
| d. The general practitioner | | 11.9% (13) | 45.9% (50) | 43.1% (47) | 33.0% (36) | 28.4% (31) | | 38.5% (42) | 24.7% (27) |
| e. The child neuropsychiatrist | | 53.2% (58) | 27.5% (30) | 2.8% (3) | 2.8% (3) | 1.8% (2) | | 1.8% (2) | 1.8% (2) |
| f. The neurologist and/or headache specialist | | 34.9% (38) | 68.8% (75) | 84.4% (92) | 85.3% (93) | 82.6% (90) | | 88.1% (96) | 75.2% (82) |
| g. The gynecologist | | 0.9% (1) | 4.6% (5) | 51.4% (56) | 67.0% (73) | 77.1% (84) | | 45.0% (49) | 2.8% (3) |
| h. The oncologist | | 0.0% (0) | 0.0% (0) | 0.0% (0) | 0.9% (1) | 0.0% (0) | | 0.0% (0) | 75.2% (82) |
| i. Other | | 0.9% (1) | 0.0% (0) | 0.9% (1) | 1.83% (2) | 0.9% (1) | | 1.8% (2) | 2.8% (3) |

| **Considering a patient in pediatric and adolescent age: Indicate the level of agreement with the following statement** | | | | | |  |
| --- | --- | --- | --- | --- | --- | --- |
|  |  | | | Agreement, % (n) | |  |
| In the management of migraine in pediatric and adolescent patients | Q16. It is crucial to investigate the social aspects that may trigger migraine attacks, especially during the transition from pre- to post-puberty (n=109) | | | 95.4% (104) | |  |
|  | Q17. The first intervention is socio-behavioral to prevent episodes and/or reduce their frequency (n=107) | | | 92.5% (99) | |  |
|  | Q18. A combined intervention (socio-behavioral and pharmacological) is necessary if the socio-behavioral intervention alone has not yielded improvements (n=107) | | | 93.5% (100) | |  |
|  | Q19. The psychologist is involved only if conditions of stress or anxiety are hypothesized (n=106) | | | 75.5% (80) | |  |
|  | Q20. The psychiatrist is involved only if a psychopathological disorder is hypothesized (n=105) | | | 90.5% (95) | |  |
|  | Q21. It is necessary to increase awareness among parents regarding the pathology and migraine symptoms (n=105) | | | 100% (105) | |  |
| **Considering an adult patient with menstrual migraine: Indicate the level of agreement with the following statement** | | | | | |  |
|  |  | | | Agreement, % (n) | |  |
| In the management of adult patients with menstrual migraine or menstruation-related migraine | Q22. Specialized consultation is necessary only in the case of prolonged and intense symptoms (n=102) | | | 44.2% (45) | |  |
|  | Q23. The treatment of migraine should be managed only by a neurologist and/or headache specialist (n=102) | | | 76.5% (78) | |  |
| **Considering an adult patient undergoing contraceptive therapy: Indicate the level of agreement with the following statement** | | | | | |  |
|  |  | | | Agreement, % (n) | |  |
| In the management of migraine in adult women requiring contraceptive therapy | Q24. The presence of migraine and aura should be carefully considered (n=101) | | | 99.0% (100) | |  |
|  | Q25. The choice of contraceptive type is based on the presence of risk factors for ischemia (n=99) | | | 96.0% (95) | |  |
|  | Q26. A consultation between a gynecologist and a neurologist/headache specialist is necessary to confirm the diagnosis of migraine with aura (n=97) | | | 76.3% (74) | |  |
|  | Q27. The actual thrombotic risk in each patient should be routinely assessed (e.g., screening for thrombophilia) before choosing a contraceptive (n=96) | | | 89.6% (86) | |  |
| **Considering an adult patient who is pregnant or breastfeeding: Indicate the level of agreement with the following statement** | | | | | |  |
|  |  | | | Agreement, % (n) | |  |
| In the management of migraine in adult women during pregnancy or breastfeeding | Q28. The presence of migraine should be noted during the initial gynecological visit (n=96) | | | 100% (96) | |  |
|  | Q29. Special attention should be paid to the onset of new-onset migraine during pregnancy (n=96) | | | 100% (96) | |  |
|  | Q30. The anti-migraine medications considered safe for both the mother and the fetus can always be administered (n=95) | | | 71.6% (68) | |  |
|  | Q31. Consultation with a neurologist and/or headache specialist is necessary for the treatment of migraine (n=94) | | | 96.8% (91) | |  |
|  | Q32. Acupuncture can be suggested as a therapy to be carried out either as a replacement or in combination with pharmacological treatment (n=94) | | | 92.6% (87) | |  |
| **Considering an adult patient undergoing assisted reproduction: Indicate the level of agreement with the following statement** | | | | | |  |
|  |  | | | Agreement, % (n) | |  |
| In the management of migraine in adult women undergoing assisted reproduction | Q33. The presence of migraines should be carefully considered before undergoing assisted reproduction techniques (n=94) | | | 97.8% (92) | |  |
|  | Q34. The gynecologist should consider the administration of alternative (lighter) hormonal stimulation protocols to reduce migraine pain (n=94) | | | 86.2% (81) | |  |
|  | Q35. A consultation with a neurologist and/or headache specialist is necessary for the treatment of migraine (n=94) | | | 97.9% (92) | |  |
|  | Q36. Psychological support is recommended (n=94) | | | 87.2% (82) | |  |
| **Considering a patient in menopause: Indicate the level of agreement with the following statement** | | | | | |  |
|  |  | | | Agreement, % (n) | |  |
| In the management of migraine in menopausal women | Q37. The type of HRT should be chosen according to the type of migraine and after a careful clinical evaluation of the patient (n=94) | | | 100% (94) | |  |
|  | Q38. The peri-menopausal phase should be carefully monitored to avoid strong hormonal fluctuations that trigger migraine attacks (n=94) | | | 100% (94) | |  |
|  | Q39. The onset of newly emerging migraine during menopause/HRT should be carefully monitored (n=94) | | | 100% (94) | |  |
|  | Q40. Consultation with a neurologist and/or headache specialist is necessary for the treatment of migraine (n=94) | | | 96.8% (91) | |  |
| **Considering an adult patient undergoing oncological treatment: Indicate the level of agreement with the following statement** | | | | | |  |
|  |  | | | Agreement, % (n) | |  |
| In the management of migraine in adult women undergoing oncological treatment | Q41. The worsening of migraine symptoms during treatment should be monitored (n=93) | | | 100% (93) | |  |
|  | Q42. Active collaboration between the oncologist and neurologist/headache specialist is necessary (n=93) | | | 98.9% (92) | |  |
| **General considerations on follow-up management: Indicate the level of agreement with the following statement** | | | | | |  |
|  |  | | | Agreement, % (n) | |  |
| The management of migraines in the various stages of a woman's life | Q43. Must ensure the continuity of care (follow-up visits, renewal of treatment plans) (n=93) | | | 98.9% (92) | |  |
|  | Q44. Must take place in headache centers (hospital-based or university-affiliated) or headache clinics (n=93) | | | 91.4% (85) | |  |
|  | Q45. Must take place at the local or community level (n=93) | | | 91.4% (85) | |  |
|  | Q46. Must be shared between headache centers (hospital-based or university-affiliated) or headache clinics, and the local community (n=93) | | | 97.9% (91) | |  |
|  | Q47. Must involve the role of the caregiver (n=93) | | | 80.7% (75) | |  |
|  | Q48. Must include the use of a dedicated digital platform to enhance the exchange of information and documents about patients among various medical professionals (n=93) | | | 86.0% (80) | |  |
|  | Q49. Must include the support of telemedicine (n=93) | | | 84.9% (79) | |  |
| **Training and education of the pharmacist: Indicate the level of agreement with the following statement** | | | | | |  |
|  |  | | | Agreement, % (n) | |  |
| Training and updating on the management of migraine patients for the pharmacist | Q50. Are necessary (n=93) | | | 94.6% (88) | |  |
|  | Q51. Must be mandatory (n=93) | | | 84.9% (79) | |  |
|  | Q52. Must include instructions for the use of tools (e.g., ID-migraine) to be used for categorizing headaches (migraine or other types) (n=93) | | | 71.0% (66) | |  |
| **Training and education of the general practitioner and pediatrician: Indicate the level of agreement with the following statement** | | | | | |  |
|  |  | | | Agreement, % (n) | |  |
| Training and updating on the management of migraine patients for the general practitioner and pediatrician | Q53. Are necessary (n=92) | | | 100% (92) | |  |
|  | Q54. Must be mandatory (n=92) | | | 96.7% (89) | |  |
|  | Q55. Must include instructions for the use of tools (e.g., ID-migraine) to be used for categorizing headaches (migraine or other types) (n=91) | | | 94.5% (86) | |  |
| **Training and education of the specialized physician: Indicate the level of agreement with the following statement** | | | | | |  |
|  |  | | | Agreement, % (n) | |  |
| Training and updates on the management of migraine patients for other specialists (neurologists, gynecologists, oncologists, etc.) | Q56. Are necessary (n=91) | | | 98.9% (90) | |  |
|  | Q57. Must be mandatory (n=91) | | | 91.2% (83) | |  |
|  | Q58. Must include instructions for the use of tools (e.g., ID-migraine) to be used for categorizing headaches (migraine or other types) (n=91) | | | 91.2% (83) | |  |
|  | Q59. Must be implemented given the lack of scientific evidence supporting the optimal management of patients with migraines in specific settings (assisted reproduction, patients with migraine undergoing oncological treatment) (n=91) | | | 97.8% (89) | |  |
| **Awareness of the condition for patients: Indicate the level of agreement with the following statement** | | | | | |  |
|  |  | | | Agreement, % (n) | |  |
| Among the initiatives to promote awareness of migraine in women, the following should be encouraged | Q60. The use of social media platforms (n=91) | | | 90.1% (82) | |  |
|  | Q61. The number of 'headache awareness days' throughout the year (n=91) | | | 85.7% (78) | |  |
|  | Q62. The availability of questionnaires in pharmacies (e.g., ID-migraine) (n=91) | | | 85.7% (78) | |  |
|  | Q63. All of the abovementioned, as the current ones in place are insufficient (n=91) | | | 93.4% (85) | |  |
| **Awareness of the condition for patients: Please answer the following questions** | | | | | |  |
|  | Yes, % (n) | | | | |  |
| Q64. Would you consider useful to have a pamphlet for women that highlights the importance of the symptoms and the phases of the patient’s journey (n=91) | | 93.4% (91) | | | |  |
|  | Migraine as a stigma, % (n) | | The neglect of the symptom, % (n) | Reference medical figures, % (n) | Socio-economic disease burden, % (n) | Other, % (n) |
| Q65. In your opinion, which awareness themes regarding migraine pathology in women should be further explored? (possible multiple answers) (n=91) | 34.1% (31) | | 67.0% (61) | 83.5% (76) | 56.0% (51) | 8.9% (8) |

**
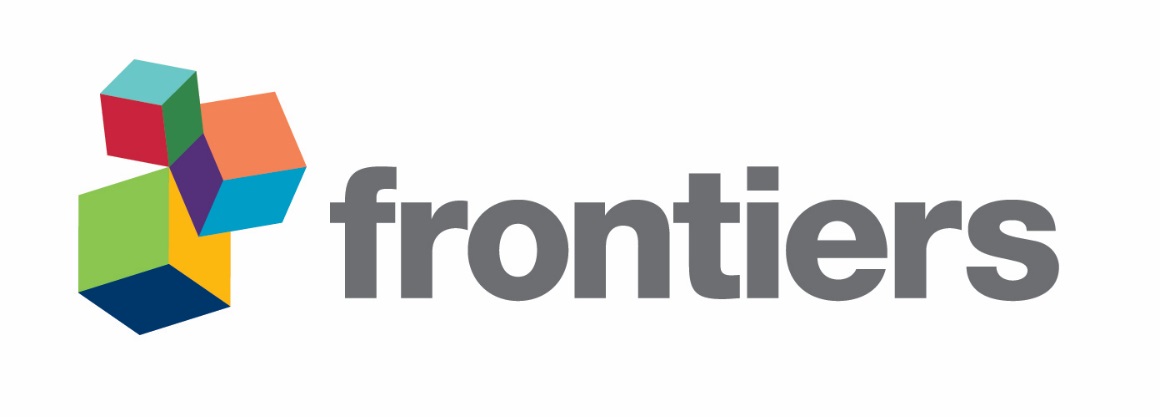
**
